# Amino Acids—Potential Biomarkers of Histological Features for MASLD in Pediatric Obesity

**DOI:** 10.3390/ijms27083596

**Published:** 2026-04-17

**Authors:** Diana Zamosteanu, Nina Filip, Ludmila Lozneanu, Simona Eliza Giusca, Oana Viola Badulescu, Mihaela Pertea, Alexandru Filip, Carmen Ungureanu, Eugenia Morosan, Elena Cojocaru

**Affiliations:** Grigore T. Popa University of Medicine and Pharmacy, 700115 Iasi, Romania; zamosteanu.diana@d.umfiasi.ro (D.Z.); ludmila.lozneanu@umfiasi.ro (L.L.); simona-eliza.giusca@umfiasi.ro (S.E.G.); oana.badulescu@umfiasi.ro (O.V.B.); mihaela.pertea@umfiasi.ro (M.P.); alexandru-filip@umfiasi.ro (A.F.); carmen.ungureanu@umfiasi.ro (C.U.); eugenia.morosan@umfiasi.ro (E.M.); elena2.cojocaru@umfiasi.ro (E.C.)

**Keywords:** MASLD, amino acids, biomarkers, children, obesity

## Abstract

Metabolically-dysfunction-associated steatotic liver disease (MASLD) represents the most common chronic liver disease in the pediatric population, and its prevalence has doubled over the past decade. The etiology is multifactorial, including genomic risk factors, perinatal and developmental or behavioral factors. Still, many cases of MASLD are associated with being overweight and obesity, particularly in children who have poor dietary habits and sedentary lifestyles that contribute to excessive weight gain. Given the progressive and heterogeneous nature of MASLD, early identification of high-risk patients before the development of severe liver disease is a major clinical priority. Recent studies indicate that disorders of amino acid metabolism are closely linked to both obesity and MASLD, reflecting profound alterations in systemic metabolic homeostasis. The reported data sustain significant changes in circulating amino acid profiles, particularly elevated levels of branched-chain amino acids (BCAAs) and aromatic amino acids. These alterations are thought to reflect fundamental metabolic disturbances, including insulin resistance, compromised mitochondrial activity, and altered hepatic lipid metabolism. Consequently, alterations in amino acid metabolism have been proposed as potential biomarkers for disease progression and metabolic dysfunction in MASLD. This review aims to evaluate the correlation between the amino acid profile and histological changes in pediatric MASLD, including steatosis, steatohepatitis, and fibrosis.

## 1. Introduction

Overweight and obesity in children have become a major health concern, with substantial and long-term socio-economic costs through the early and later associated modifications [1,2,3]. Inflammatory, metabolic, and hormonal alterations contribute to the development of multiple comorbidities, including type 2 diabetes mellitus (T2DM), dyslipidemia, hypertension, metabolic dysfunction-associated steatotic liver disease (MASLD), obstructive sleep apnea, and polycystic ovary syndrome [4,5,6,7].

The global obesity epidemic has significant metabolic consequences, including the rising prevalence of pediatric MASLD, from 7.6% in 2015 to 14.3% in 2024. Understanding the link between MASLD and obesity is essential for prevention and early intervention [8]. Metabolically-dysfunction-associated steatotic liver disease, previously referred to as non-alcoholic fatty liver disease (NAFLD), has become a major health issue in the pediatric population, as a result of the increasing prevalence of overweight and obesity [7]. In 2023, an international consensus statement modified this nomenclature of fatty liver disease [9]. An analysis of this revised terminology is particularly relevant in pediatric populations, in whom alcohol intake is not a defining factor, and metabolic dysfunction represents the primary driver of steatotic liver disease. Additionally, the stigmatizing terms “fatty” and “non-alcoholic” were excluded [10,11]. This change in nomenclature does not alter the clinical and pathological criteria used for diagnosis or disease staging [12]. Additionally, this term-reconceptualization highlights the metabolic mechanism and has relevant implications for early diagnosis and intervention, particularly in children [13]. According to the current guideline, MASLD diagnosis requires the presence of hepatic steatosis, inflammation, and hepatocellular lesion, demonstrated by histology in the context of metabolic dysfunction, rather than alcohol-related injury.

Liver biopsy with histopathological assessment of hepatic tissue remains the reference standard recommended by current guidelines to establish the presence and severity of MASLD. Although it is recommended, liver biopsy is not practical in many healthcare settings due to its invasiveness, cost, and risk of complications [14]. Thus, the use of standardized and validated non-invasive tests for the pediatric population is essential for assessing disease severity and monitoring disease progression.

Amino acids have been investigated as potential markers of liver injury and disease progression because they are involved in various hepatic metabolic pathways and pathophysiological processes [15,16,17,18]. Among these, alterations in circulating amino acid profiles have emerged as important indicators of metabolic dysfunction. In particular, increased levels of branched-chain amino acids (BCAAs), including leucine (Leu), isoleucine (Ile), and valine (Val), have been consistently linked to obesity and insulin resistance. These metabolic changes are thought to contribute to impaired glucose and lipid metabolism, thereby promoting the development and progression of MASLD [4]. Moreover, disturbances in amino acid metabolism may reflect underlying mitochondrial dysfunction, oxidative stress, and alterations in hepatic energy metabolism, which are key mechanisms involved in MASLD pathogenesis [19]. However, the extent to which these metabolic changes reflect specific histopathological stages in pediatric MASLD remains unclear. Throughout this review, the updated nomenclature MASLD, MASL (metabolic dysfunction-associated steatotic liver), and MASH (metabolic dysfunction-associated steatohepatitis) is adopted, while legacy terms NAFLD/NASH are used only when required for consistency with the original literature.

The results of the presented studies are limited and heterogeneous, and the relationship between amino acid profile and liver histological changes in children is not fully elucidated. Therefore, the main objective of this review is to critically synthesize current evidence on the relationship between amino acid metabolism and histological features of pediatric MASLD, with a focus on their potential as non-invasive biomarkers.

## 2. Obesity-Induced Low-Grade Chronic Inflammation

Obesity is known as a state of low-grade chronic inflammation, mediated by sustained activation of the innate immune system, which leads to disruption of metabolic homeostasis [20,21]. Visceral adipose tissue plays a truly important role in this process, functioning not only as an energy reservoir but also as an active endocrine organ that secretes adipokines and pro-inflammatory cytokines. These mediators affect insulin signaling pathways, which can promote reduced cellular glucose uptake and the development of insulin resistance [22]. Excessive visceral adiposity is strongly associated with systemic insulin resistance, initially affecting skeletal muscle and subsequently involving adipose tissue and the liver. At the hepatic level, this is characterized by inadequate suppression of gluconeogenesis and impaired glucose utilization [23,24]. Under physiological conditions, insulin promotes glycogen synthesis, stimulates lipogenesis, and inhibits gluconeogenic pathways. In states of insulin resistance, these regulatory effects are attenuated, requiring elevated levels of circulating insulin to maintain glucose homeostasis [25]. Insulin signaling is mediated predominantly through insulin receptor substrate 1 (IRS1) and insulin receptor substrate 2 (IRS2), which mediate insulin receptor to downstream intracellular signaling pathways [26]. Activation of IRS-dependent signaling through the PI3K–AKT pathway suppresses hepatic gluconeogenesis and promotes glycogen synthesis, while parallel RAS–MAPK/ERK signaling regulates hepatocyte growth and survival. Impairment of hepatic IRS signaling results in inadequate suppression of glucose production and contributes to hepatic insulin resistance, a central mechanism in the pathogenesis of MASLD [23,24,27].

The adaptive modifications of adipose tissue promote lipolysis, which can lead to increased circulating free fatty acids (FFAs) and intracellular lipid accumulation [4,28]. At the hepatic level, the imbalance between the excessive influx of free fatty acids from endogenous stores, in the context of insulin resistance, and the overload of beta-oxidation will lead to the onset of mitochondrial dysfunction and the overproduction of reactive oxygen species (ROS). Mitochondrial dysfunction represents a key element in the pathophysiology of the metabolic syndrome associated with MASLD [29]. The depletion of mitochondria and other intracellular organelles, such as the endoplasmic reticulum and peroxisomes, will sustain the apoptotic cascade and cell death [30]. Cells that play a major role in perpetuating inflammation and activating fibrosis are represented by liver macrophages, known as Kupffer cells, which are part of the innate immune system. The activation of Kupffer cells, with the intracellular accumulation of cholesterol, marks the onset of inflammation in liver pathology and promotes the progression of MASLD to steatohepatitis [31]. Hepatocyte lipotoxicity, accentuated by excess FFAs, will increase the production of pro-inflammatory cytokines, and activated Kupffer cells will release cytokines, chemokines, nitric oxide, and ROS. These signals help recruit leukocytes such as neutrophils, monocytes, and natural killer T (NKT) cells, which further support inflammation in the liver. Furthermore, adipose tissue expansion leads to increased levels of pro-inflammatory cytokines such as tumor necrosis factor-α (TNF-α), interferon-γ (IFN-γ), interleukin-1β (IL-1β), and interleukin-6 (IL-6), as well as adipokines including leptin, resistin, retinol-binding protein 4 (RBP-4), angiopoietin-like protein 2 (ANGPTL2), and lipocalin-2, have been shown to play important roles in the development of insulin resistance and inflammation [4,6,32,33]. Concurrently, obesity is characterized by activation and proliferation of CD4^+^ and CD8^+^ T cells within adipose tissue depots, contributing to sustained inflammatory responses [34].

In obese patients with insulin resistance, caloric intake fails to appropriately suppress glycogenolysis. Despite caloric consumption and the postprandial rise in blood glucose levels, hepatic glucose production persists, leading to glucotoxicity. This process becomes a self-perpetuating cycle that further exacerbates insulin resistance [4,35]. These modifications promote hepatic lipid accumulation, inflammation, and endoplasmic reticulum stress [36].

## 3. Management of Pediatric MASLD

MASLD is defined as excessive hepatic lipid accumulation in association with one or more cardiometabolic risk factors, without alcohol consumption or alternative causes of liver injury. In pediatric practice, these cardiovascular indicators/criteria comprise: body mass index (BMI) ≥ 85th percentile (BMI z-score ≥ +1), blood pressure ≥ 130/80 mmHg, fasting glucose ≥ 100 mg/dL, plasma triglycerides ≥ 100 mg/dL (age below 10 years) or ≥150 mg/dL (age 10 years or above), and plasma HDL cholesterol ≤40 mg/dL [37]. Overweight and obesity represent a cardiometabolic risk factor, and children with increased BMI who present hepatic steatosis ≥5% are diagnosed with MASLD [38]. According to the NASPGHAN Clinical Practice Guideline, MASLD diagnosis requires the presence of chronic hepatic steatosis in the context of metabolic dysfunction, rather than alcohol-related injury.

MASLD screening is recommended for the high-risk population, including obese and overweight children with cardiometabolic risk factors. The optimal age is not established, but the actual guide recommends beginning between the ages of 9 and 11 years. Non-invasive, serological, or imaging tests have not shown effectiveness in identifying children with MASLD [39]. Recent data highlight the need for an integrated screening strategy to improve the early identification of MASLD in this vulnerable population [40]. Conversely, in adults with MASLD, several non-invasive tests, including blood-based biomarkers (Aspartate Aminotransferase to Platelet Ratio Index (APRI), Fibrosis Indices (FIB-4), and the Enhanced Liver Fibrosis (ELF)) and imaging modalities (Ultrasound (US), Vibration-Controlled Transient Elastography (VCTE), and Magnetic Resonance Elastography (MRE)), are available and widely used in clinical practice for screening, diagnosis, and disease monitoring. However, none replace liver biopsy [41]. The performance and validation of these adult-derived strategies cannot be directly extrapolated to pediatric populations, and the applicability remains limited. According to practice statements on MASLD evaluation in children, alanine aminotransferase (ALT) is currently recommended for screening and evaluation of hepatic involvement. ALT, although inexpensive and minimally invasive, demonstrates limited diagnostic performance in pediatric MASLD, with only moderate sensitivity (80%) and poor specificity (26%). Nevertheless, ALT is not a reliable marker of liver disease severity, as it mainly reflects steatohepatitis and can be normal despite advanced fibrosis [39].

Multiple scoring systems for MASLD integrating anthropometric measurements and serum biomarkers have been proposed for MASLD assessment. A large cohort study of pediatric patients with biopsy-confirmed MASLD reports the modest diagnostic accuracy of these scores in discriminating fibrosis stages and indicates they are not superior to serum ALT alone [42].

Non-invasive imaging modalities are available for the evaluation of hepatic steatosis in children, each having advantages and limitations. B-mode ultrasound (US) is easily accessible and inexpensive, but has limited diagnostic performance in the pediatric population due to suboptimal sensitivity and specificity, particularly in children with mild steatosis (involving ≤33% of hepatocytes). Additionally, increased liver echogenicity may be caused by other conditions, such as hepatitis or infiltrative liver diseases [43,44]. VCTE shows a reasonable balance between diagnostic accuracy, cost-effectiveness, and practical and logistical considerations. VCTE provides two distinct parameters: the controlled attenuation parameter (CAP), which estimates hepatic steatosis, and liver stiffness measurement (LSM), which reflects hepatic fibrosis. Pediatric studies have demonstrated good feasibility and a strong association between CAP and histological steatosis. Despite these advantages, pediatric cut-off values remain variable across studies [38,45,46]. Magnetic resonance imaging (MRI) provides the highest accuracy for quantifying hepatic steatosis and fibrosis and differentiating between simple steatosis and NASH. Their use in children is limited by high costs, restricted availability, the potential need for sedation in uncooperative patients, and the requirement for specialized radiological expertise [29,47,48]. Hepatic steatosis is occasionally identified on computed tomography (CT) performed for other clinical indications, but CT is not routinely used for MASLD screening in children due to radiation concerns [39]. While non-invasive diagnostic methods have expanded considerably and offer important advantages, achieving diagnostic accuracy comparable to liver biopsy remains an area requiring further improvement.

Liver biopsy with histopathological assessment of hepatic tissue remains the gold standard recommended by current guidelines to establish the presence and severity of MASLD [39]. However, liver biopsy is not practical in many healthcare settings because of its high cost, invasive nature, and risk of complications—such as pain, bleeding, and injury to adjacent organs—which reduces the feasibility of its routine use, particularly in pediatric patients [49]. In addition, the diagnostic accuracy of liver biopsy is limited by sampling variability due to the focal and heterogeneous distribution of disease within the liver, as well as inter- and intra-observer variability in histological interpretation, which may introduce diagnostic bias [42,50].

Despite the preliminary and exploratory use of medications and supplements, no medications have been approved for pediatric MASLD. In studies evaluating the GLP-1 receptor agonists (GLP-1 RAs) for obesity and T2DM, additional benefits beyond glycemic control and weight loss have been observed, including improvements in hepatic biomarkers and metabolic parameters in pediatric MASLD. These findings suggest potential therapeutic effects, but additional data are required to define the impact of GLP-1 RAs on steatosis and fibrosis in the pediatric population [51,52]. Furthermore, bariatric surgery is not considered a disease-specific treatment due to insufficient outcome data in adolescents with MASLD [53].

## 4. Amino Acids Changes Across Histopathological Stages of MASLD

### 4.1. Histopathological Features of Pediatric MASLD

Based on histology, MASLD includes a spectrum ranging from liver steatosis (MASL, formerly classified as NAFL, non-alcoholic fatty liver) to metabolic dysfunction-associated steatohepatitis (MASH, previously termed NASH, non-alcoholic steatohepatitis), which can progress to fibrosis and cirrhosis [41,54]. MASH is the most significant form of MASLD, and develops when chronic fat accumulation determines cell injury and is characterized by the presence of hepatocyte ballooning, lobular inflammation, macrovesicular steatosis, and perisinusoidal fibrosis [55]. The histopathological features of MASH in children may differ from those observed in adult patients. In adults, MASH is characterized by hepatic steatosis, lobular inflammation (mononuclear cells or polymorphonuclear cells or both), and hepatocyte lesion (ballooning), in the presence or absence of fibrosis. Other microscopical features comprise Mallory Denk bodies, iron accumulation within hepatocytes or reticuloendothelial cells, ductular reaction, megamitochondria within hepatocytes, and glycogenated hepatocytes displaying vacuolated nuclei [56].

**Figure 1:**
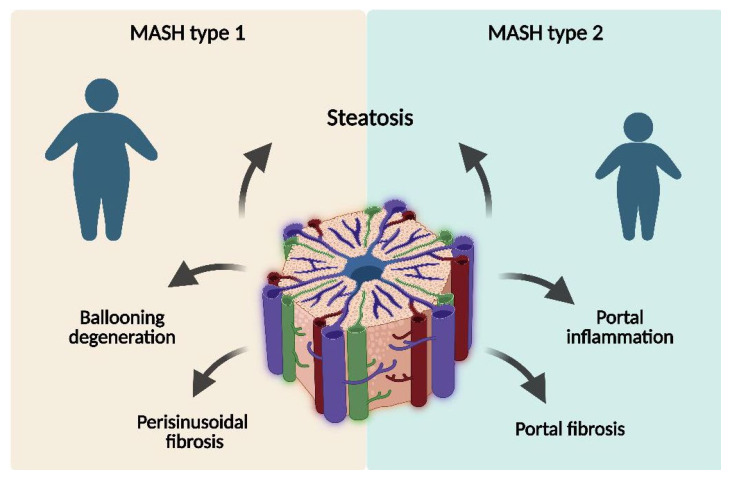
Schematic representation of Type 1 (adult-like) and Type 2 (pediatric) metabolic dysfunction-associated steatohepatitis (MASH). Created with BioRender.com, Diana Zamosteanu (2026), https://BioRender.com/1hdd2m9 (accessed on 21 March 2026). Type 1 is characterized by steatosis with hepatocellular ballooning and/or perisinusoidal fibrosis, whereas Type 2 shows steatosis with predominant portal inflammation and/or fibrosis.

A landmark histopathological study by Schwimmer et al. [57] described two distinct patterns of pediatric NASH (corresponding to MASH under current nomenclature)—MASH type 1 (adult-like pattern)—presence of steatosis with ballooning degeneration and/or perisinusoidal fibrosis without portal involvement, and MASH type 2—presence of steatosis accompanied by portal inflammation and/or fibrosis in the absence of ballooning degeneration and perisinusoidal fibrosis (Figure 1). Type 2 MASH was the predominant form and was observed more frequently in younger patients, who had a higher degree of obesity. In type 2 MASH, the central vein region was consistently spared, with no evidence of hepatocellular lesions in zone 3, and portal inflammation was predominantly lymphocytic [57]. The typical adult pattern of MASH is seen predominantly in adolescents, while younger pre-adolescents and children often exhibit an alternate pattern of progressive liver disease [58].

Recent data sustain overlapping features, suggesting that pediatric MASLD represents a continuum of histopathological change [59,60,61]. Carter et al. [62] demonstrated that portal-based injury, found in children, is frequently associated with adult-type histological features. The heterogeneity of histological patterns in pediatric MASH may reflect a pathological process that initially involves zone 1 and subsequently progresses toward the adult-type centrilobular pattern during the natural history of the disease [63]. Use of MASH type 1 and MASH type 2 may be impractical because many cases present overlapping features, while intralobular and portal findings are positively associated [64].

MASH severity can be appreciated through histological grading systems. Brunt et al. [65] introduced a staging system considering the steatosis, ballooning degeneration, inflammation, and fibrosis patterns [65]. This system served as the basis for the development of the NAFLD Activity Score (NAS), a semi-quantitative system for histological evaluation of MASLD, which was developed and validated by David E. Kleiner et al. [66] within the MASH Clinical Research Network (MASH CRN). This grading system appreciates fibrosis independently using a staging system that is suitable for assessing the response to treatment and the histological alterations found in clinical investigations. NAS criteria include lobular inflammation, but not portal inflammation—a feature frequently observed in the pediatric population. This may underestimate the degree of inflammation, especially if the inflammatory infiltrate is located only in this area [66]. Bedossa et al. [67] proposed a scoring system similar to that of the MASH CRN, the Steatosis, Activity, and Fibrosis (SAF) score, which includes an algorithm for classifying MASLD into MASL and MASH based on histological findings. However, its application in pediatric populations remains limited and requires future validation studies [58,67]. Histologically characterized pediatric MASLD cohorts remain limited, and available data are derived from relatively small studies employing different diagnosis approaches. While similar metabolic alterations have been described in adult MASLD, pediatric disease exhibits distinct histopathological features, including periportal steatosis and portal-based inflammation.

### 4.2. Amino Acids in MASLD

**Figure 2:**
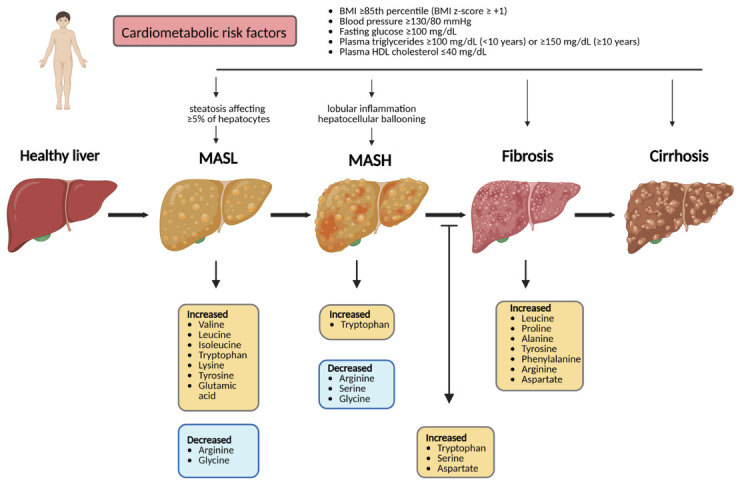
Schematic representation of liver disease progression associated with amino acid alterations in MASLD. Created with BioRender.com, Diana Zamosteanu (2026), https://BioRender.com/23vyj4q (accessed on 21 March 2026).

BCAAs and aromatic acid levels are dysregulated in adults with MASLD relative to subjects with reduced liver lipid accumulation. These findings provide evidence for a mechanistic involvement of amino acid metabolism in MASLD development and progression (Figure 2) [68,69]. For example, in adults, the study conducted by Pérez-Díaz et al. [70], in obese patients undergoing bariatric surgery, reported changes in amino acid concentrations in individuals diagnosed with MASH. Specifically, the authors reported increased hepatic levels of arginine (Arg), glycine (Gly), and cystine in patients with MASH compared with those with simple steatosis. The levels of the amino acids lysine (Lys) and Arg were positively associated with histopathological severity, as well as with biochemical markers of liver injury. Imbalance in hepatic amino acid metabolism may contribute to the pathophysiology of MASLD [70]. Similar metabolic alterations have been observed in children, where disrupted amino acid pathways, including BCAAs metabolism, were associated with MASLD [71,72].

Khusial et al. [73], in a study conducted on the pediatric population, reported amino acids serine (Ser), Leu, Ile, and tryptophan (Trp) as the main metabolites associated with MASLD. Decreased amino acid Ser and increased BCAAs and aromatic amino acids indicate their disturbed metabolism in pediatric MASLD. Decreased Ser concentrations were inversely associated with the severity of MASLD in adults. A possible explanation would be the increased hepatic consumption for glutathione biosynthesis [73]. A non-invasive MASLD screening panel, consisting of clinical and metabolic features, was established by Huneault et al. [37] in a cohort of 130 children with MASLD diagnosed by imaging and/or biopsy. The composite model demonstrates disturbances in the metabolic pathways. BCAAs—Leu/Ile were significantly elevated in children with MASLD, representing the most significant amino acid predictor of MASLD [37]. Trp levels were also increased, supporting prior evidence of associations between aromatic amino acid perturbations, insulin resistance, and metabolic inflammation [74]. Although Ser demonstrates a modest statistical significance across analyses. Collectively, these findings highlight the role of the altered amino acid metabolism—particularly BCAA and aromatic acids—as a key metabolic feature of pediatric MASLD [37].

In another integrative analysis by Huneault et al. [75], including 514 children with biopsy-proven MASLD from three NASH Clinical Research Network studies, MASLD was classified in three subtypes based on clinical and metabolic findings: early-mild, cardiometabolic, and inflammatory-fibrotic groups. The inflammatory-fibrotic group was characterized by elevated liver enzymes, steatohepatitis, and advanced fibrosis. Distinct amino acid alterations were observed across histological stages, particularly in relation to inflammation and fibrosis. While elevated BCAAs, especially Leu and Ile, appeared to be more closely associated with systemic metabolic dysfunction—reflected by higher levels of BCAAs in cardiometabolic subtype, along with dyslipidemia and insulin resistance—aromatic amino acids perturbations were correlated with distinct features of MASLD. The inflammatory-fibrotic metabotype presented disruptions in the kynurenine pathway, including Trp, kynurenine, serotonin, and indole derivatives; upregulated levels demonstrated positive correlations with ALT, AST, and fibrosis stage [75]. These findings align with other studies sustaining the strong link between activation of Trp catabolism and hepatic inflammation and fibrogenesis [74]. Circulating Trp-derived metabolites are also increased in pediatric obesity and are associated with systemic low-grade chronic inflammation [76].

Pathway enrichment analysis further detected significant alterations in Gly, Ser, and threonine (Thr) metabolism within the inflammatory-fibrotic metabotype, comprising elevated hydroxyproline levels, consistent with increased collagen turnover [75,77,78]. In addition, metabolites related to pantothenate and CoA biosynthesis, such as aspartate (Asp), were also significantly higher in this group. Collectively, BCAAs elevation predominantly reflects systemic metabolic stress, while dysregulation of Trp metabolism appears to be a key metabolic pathway of steatohepatitis and fibrosis in pediatric MASLD [75].

The results reported by Chae et al. [71] highlight a distinct metabolomics profile in children diagnosed with MASLD. Participants were classified based on MASLD status, determined through liver ultrasound assessment. Circulating levels of BCAAs (Val, Leu, Ile), as well as Lys, tyrosine (Tyr), and glutamic acid (Glu), were significantly increased in overweight children with MASLD compared to overweight controls. The results agree with previous studies, which reported low Gly concentrations in the context of MASLD [71].

However, these studies evaluated differences based on the presence of MASLD, without an explicit stratification by grades of inflammation or fibrosis. Thus, these findings indicate a metabolic disruption of amino acids in pediatric MASLD and support a mechanistic involvement of an altered amino acid profile.

### 4.3. Steatosis

Hepatic triglyceride accumulation in MASLD is determined by excessive de novo lipogenesis, a process dependent on insulin signaling. In pediatric patients, steatosis is predominantly located in the periportal area, a change that suggests a different causal mechanism than those related in adults [79].

Kordy et al. [80] evaluated the oral and fecal microbiome and plasma metabolites from 241 predominantly Latin children with MASLD, clinically or biopsy-confirmed diagnosis. As expected for pediatric MASLD populations, most affected participants were overweight or obese, with higher BMI percentiles and markers of insulin resistance observed in those with more advanced histologic disease. The metabolic signature of steatosis mainly included lipid metabolites, but also compounds derived from amino acid metabolism, especially glutathione. The presence of metabolites associated with the γ-glutamyl cycle indicates early activation of oxidative stress mechanisms and glutathione turnover in the context of hepatic lipid accumulation [80].

In the study conducted by Goffredo et al. [81], the diagnosis of MASLD was established through MRI, a reliable non-invasive technique for assessing hepatic steatosis. Obese adolescents with MASLD had significantly elevated circulating BCAAs (Val, Leu, and Ile), as well as Trp, Lys, and Glu, along with increased levels of carnitine esters and long-chain phosphatidylcholines. Elevated Val levels were correlated with higher hepatic fat accumulation, and the baseline was found to be predictive of longitudinal increases in liver fat during the two-year follow-up period [81].

Jin et al. [82] applied a metabolomics-based approach to profile 9583 metabolites in 39 obese children with MASLD imaging diagnosed. Adolescents with an increased degree of steatosis exhibited a modified metabolomics profile, including disturbances across multiple amino acid concentrations, such as Tyr and Trp, BCAAs, as well as Gly and Ser. Tyr metabolism exhibited the most pronounced disruption and was positively correlated with the severity of steatosis. Thus, these findings highlight a broad dysregulation of amino acid homeostasis in pediatric MASLD and suggest that the Tyr metabolic pathway may represent a key contributor to disease progression, necessitating a comprehensive mechanistic investigation [82].

In a cohort of severely obese children and adolescents aged 9–19 years, Lischka et al. [83] demonstrated that plasma BCAAs were significantly elevated in individuals with MASLD and were positively correlated with MRI-measured intrahepatic lipid content. Specifically, Val, Leu, and Ile were significantly elevated in participants with MASLD compared to those without steatosis, indicating a close link association between BCAAs levels and the degree of hepatic lipid accumulation in severely obese youth. The study did not focus on histological severity, but instead focused on quantitative liver fat fraction, measured by MRI-PDFF. Higher circulating BCAAs concentrations were associated with progressively higher MRI-PDFF values, supporting their relationship with progressive hepatic steatosis. Furthermore, the authors developed a BCAAs-based metabolic score that exceeded other indices of hepatic fat content prediction, achieving a high diagnostic accuracy for identifying severe steatosis [83].

Overall, these data indicate that hepatic steatosis in MASLD in children is associated with a characteristic metabolic profile, marked by elevated levels of BCAAs and aromatic amino acids, as well as alterations in glycine and serine metabolism.

### 4.4. Inflammation

In the study by Kordy et al. [80], the MASH group presented modified levels of α-Ketoglutarate (α-KG), glutathione synthesis, markers of oxidative stress, methionine (Met), phosphatidylinositol, phosphatidylcholine, amino acids, Trp, sphingolipids, and concomitantly decreased levels of Ser and Gly. These changes reflect disturbances in one-carbon metabolism, mitochondrial dysfunction, and increased oxidative stress. The authors emphasize that upregulation of α-KG is one of the most important metabolite predictors of MASH [80]. α-KG represents a central metabolic pivot derived from glutamate and serves as a key link between amino acid metabolism and mitochondrial energy production. Alterations of α-KG levels are associated with increased oxidative stress, as glutamate is an essential precursor for glutathione synthesis, and its imbalance impairs cellular redox homeostasis [84]. Furthermore, α-KG supports the energetic demands of activated hepatic stellate cells, thereby contributing to their proliferation and to the fibrogenic processes characteristic of MASH [85]. A positive correlation was observed between lobular inflammation and amino acids—Trp, glutamine, vitamin B6 metabolic pathways, and metabolites related to fatty acid and lysophospholipid metabolism. In addition, Kordy et al. [80] reported a combination of 15 metabolites, including α-KG, cysteine-glutathione disulfide, and N-acetylmethionine that distinguishes MASL from MASH with 90% accuracy [80]. An integrative metabolomics network analysis of pediatric subjects with MASLD biopsy and imaging was conducted to identify metabolomics patterns corresponding to different stages of progression. Jamshidi et al. [86] revealed distinct histopathology-specific metabolic features, including a negative correlation between L-arginine and lobular inflammation [86]. Changes in amino acid metabolism, particularly increased concentrations of tryptophan, along with decreased levels of glycine and serine, contribute to inflammation and fibrosis by promoting oxidative stress and disrupting the redox balance.

### 4.5. Fibrosis

Hepatic fibrosis in MASLD develops as a consequence of chronic hepatocellular injury and inflammation. Lipotoxicity, oxidative stress, and pro-inflammatory cytokines activate hepatic stellate cells, which differentiate into myofibroblasts and produce excessive extracellular matrix constituents, especially type I and III collagen [87]. Fibrosis is the main factor of clinical events, and current results regarding progression remain controversial [88]. Adult studies revealed increased levels of BCAAs and other amino acids—Glu, glutamine (Gln), and Ala, and decreased levels of Gly in subjects with liver fibrosis [89,90,91].

Kordy [80] reported that the liver fibrosis exhibited a distinct metabolic signature, predominantly characterized by increases in sphingolipids and ceramides, as well as changes in metabolites involved in inflammatory and oxidative processes. Although amino acids were not dominant in the fibrosis model, disruptions associated with glutathione metabolism and the TCA cycle, highlighted by the increase in α-KG in MASH, suggest a metabolic context contributing to fibrosis progression. These findings are consistent with previous pediatric and adult MASLD studies, which have implicated mitochondrial dysfunction, altered energy metabolism, and redox imbalance throughout the progression from simple steatosis to steatohepatitis and fibrosis [92,93]. Importantly, when metabolomics data were integrated with gut microbiome profiling and analyzed using machine-learning approaches, the resulting models achieved high predictive accuracy for disease subtypes. This strategy highlights the potential of combined multi-omics platforms to serve as noninvasive tools for disease stratification [80]. A cross-sectional analysis including 79 children with obesity described the metabolomics phenotypes associated with two stages of MASLD progression—MASLD and MASLD with fibrosis. According to Garibay-Nieto et al. [94], MASLD is characterized by increased concentrations of ALT and decreased Arg, Gly, and tiglylcarnitine. Additionally, the fibrosis stage in MASLD correlates with a specific metabolic signature marked by increased circulating levels of proline (Pro) and alanine (Ala) and a decreased Matsuda Index. Consistent with these findings, heatmap analyses further demonstrated that children with MASLD and fibrosis exhibited a broader metabolic disturbance compared to those with isolated steatosis, including elevated concentrations of multiple amino acids—Met, Leu, Gly, Arg, Phe, ornithine, and citrulline [94]. Jamshidi et al. [86] observed a divergent association of fibrosis, including an inverse relationship with isobutyrate and a direct association with L-aspartate [86]. The correlation between fibrosis, isobutyrate, and L-glutamate has been previously reported in adults [95].

Alterations in amino acid metabolism, including increased proline, alanine, and glutamate alongside reduced glycine, contribute to fibrosis by promoting collagen synthesis, impairing antioxidant defenses, and sustaining mitochondrial dysfunction.

### 4.6. Cirrhosis

Changes in amino-acid metabolism have been consistently associated with the progression of MASLD toward fibrosis and cirrhosis [71,80,82,94]. In adult patients, early metabolic dysfunction is characterized by elevated circulating levels of BCAAs, reflecting the presence of insulin resistance. With progression to advanced liver disease and cirrhosis, this pattern tends to reverse, with decreased BCAAs levels and increased aromatic amino acids. These metabolic abnormalities reflect mitochondrial dysfunction, oxidative stress, and impaired hepatic nitrogen metabolism and may contribute to the progression of MASLD to cirrhosis [4,96,97,98,99].

Furthermore, it is considered that BCAAs, especially Val, and aromatic amino acids seem to participate in significant metabolic changes that contribute to mortality [100,101,102].

However, data regarding the amino acid profile in children with cirrhosis are still limited, and the complete characterization of these metabolic changes in the pediatric population remains insufficiently studied. Current metabolomics studies focus on identifying and characterizing early stages, aiming to describe the pathophysiological mechanisms. Nonetheless, given the rapid increase in the prevalence of MASLD among children and adolescents in recent years, it is expected that future studies will provide a more detailed characterization of the metabolic changes associated with advanced liver disease. Studies on metabolic alterations associated with MASLD are summarized in Table 1. Although a pattern of lipid metabolism dysregulation is evident in all studies, the variability of the metabolites identified implies limited comparability and potential methodological error. The reliance on noninvasive diagnostic modalities in most studies may influence the variability in classification.

Progressive alterations in amino acid metabolism, characterized by an early increase in BCAAs followed by a shift toward aromatic amino acid predominance, reflect impaired nitrogen metabolism, mitochondrial dysfunction, and oxidative stress, thereby contributing to hepatocellular injury, fibrogenesis, and progression to cirrhosis.

## 5. Limitations and Future Directions

The interpretation of the current literature is inherently limited by a number of methodological constraints that require careful analysis. Many studies rely on non-invasive imaging techniques, which may lead to a risk of misclassification, particularly when distinguishing between steatosis, steatohepatitis, and fibrosis. Furthermore, most studies rely on relatively small and clinically heterogeneous cohorts, limiting statistical power and reducing the generalizability of the results. It is important to note that body mass index, insulin resistance, pubertal stage, dietary exposure, and gut microbiome composition are considered inconsistently, thereby influencing the observed metabolic signatures. Taken together, these factors contribute to the substantial heterogeneity observed across studies and underscore the need for harmonized methodologies, well-characterized cohorts, and longitudinal studies to better define the role of amino acid metabolism in pediatric MASLD.

## 6. Conclusions

Amino acid dysregulation represents a consistent metabolic hallmark of pediatric MASLD, with branched-chain amino acids, tyrosine, tryptophan, and glycine demonstrating the most reproducible associations with disease severity. These alterations reflect underlying disturbances in insulin signaling, mitochondrial function, and redox homeostasis.

However, the current body of evidence remains heterogeneous and predominantly observational, and thus largely hypothesis-generating rather than clinically actionable. The establishment of standardized metabolomics frameworks, together with longitudinal and well-characterized pediatric cohorts, will be critical to define the clinical utility of amino acids in MASLD.

## Figures and Tables

**Table 1:** Selected studies of metabolic alterations associated with metabolic dysfunction-associated liver disease.

Author, Year, Country	Study Design	Participants	MASLD—Diagnostic Modality	Metabolites	Key Amino Acid Findings	Comments
Jin et al., 2016, USA [82]	Cross-sectional metabolomics study	39 obese adolescents aged 11–17 years, 30 with MASLD	Imaging (magnetic resonance spectroscopy, MRS)	Untargeted high-resolution plasma metabolomics (LC-MS); metabolites mainly involved in amino acid metabolism (BCAAs, aromatic amino acids)	Increased Tyr, Trp, BCAAs	Disruption of Tyr metabolism was positively correlated with the severity of steatosis.
Goffredo et al., 2017, USA [81]	Cross-sectional metabolomics study	78 obese adolescents, mean age of 13.3 ± 3.0 years, 30 with MASLD	Imaging (magnetic resonance imaging, MRI)	Targeted metabolomics (BCAAs and related metabolites)	Increased BCAAs, Trp, Lys, Glu	Increased Val concentration was associated with steatosis.
Kordy et al., 2021, USA [80]	Cross-sectional observational study	241 children aged 5–21 years, 80 with elevated BMI	Liver biopsy (MASH) and clinical diagnosis (MASL)	Untargeted plasma metabolomics profiling	Increased Trp, α-KG, Met Decreased Ser, Gly	Distinct metabolic signatures associated with steatosis and MASH.
Lischka et al., 2021, Austria [83]	Cross-sectional observational study	100 children and adolescents, 68 with MASLD and severe obesity (8–19 years)	Imaging	Targeted metabolomics of BCAAs	Increased BCAAs	Strong association between BCAAs and intrahepatic lipid content.
Chae et al., 2022, South Korea [71]	Cross-sectional metabolomics study	559 children and adolescents	Imaging (ultrasonography)	Serum metabolomics (amino acids, lipids, acylcarnitines)	Increased BCAAs, Glu, Tyr Decreased Gly	Association between amino acid profiles and MASLD presence.
Jamshidi et al., 2023, USA [86]	Cross-sectional multi-modality study	65 children and adolescents with MASLD, aged 8–17 years, 53 with obesity or severe obesity	Biopsy (histology)	Plasma metabolomics (amino acids, lipids, and bile acids)	Increased Asp Decreased Arg	Histology-specific metabolic signatures; associations with inflammation and fibrosis.
Garibay-Nieto et al., 2023, Mexico [94]	Cross-sectional metabolomics study	79 children with obesity, aged 8–16 years, 59.5% with MASLD, 21.6% with MASLD and fibrosis	Imaging (transient elastography)	Plasma metabolomics (amino acids, acylcarnitines, lipids)	Increased Pro, Ala Decreased Arg, Gly	A distinct metabolic phenotype associated with fibrosis, linked to insulin resistance.
Huneault et al., 2024, USA [37]	Cross-sectional external validation study	161 children aged 7–17 years, 130 with MASLD and elevated BMI	Biopsy or Imaging	High-resolution serum metabolomics: a panel of amino acids, phospholipids, and dihydrothymine	Increased BCAAs, Trp	Metabolomic alterations (amino acids and phospholipids) were associated with hepatic steatosis in children.
Huneault et al., 2026, USA [75]	Cross-sectional multi-omics analysis with unsupervised clustering	514 children aged 5–18 years with MASLD	Biopsy (histology)	High-resolution serum metabolomics; metabolites included uric acid, phosphatidylcholines, sphingolipids, BCAAs, kynurenine-pathway metabolites	Increase Trp, Gly, Ser, Thr, Asp	Trp/kynurenine metabolism was associated with fibrosis severity.

## Data Availability

No new data were created or analyzed in this study. Data sharing is not applicable to this article.
